# Swallowing impairment and aspiration risk in clinically stabilized patients hospitalized for acute respiratory events: a cohort-wide machine-learning analysis with COPD-specific insights

**DOI:** 10.3389/fmed.2026.1804250

**Published:** 2026-05-05

**Authors:** Anna Annunziata, Anna Michela Gaeta, Francesca Simioli, Tullio Valente, Antonietta Coppola, Maria Cardone, Raffaella Manzo, Michele Cutino, Antonella Marotta, Giuseppe Fiorentino

**Affiliations:** 1 Unit of Respiratory Pathophysiology, A.O.R.N Dei Colli, Monaldi-Cotugno Hospital, Naples, Italy; 2 Department of Respiratory Medicine, Hospital Universitario Severo Ochoa, Madrid, Spain; 3 Department of Radiology, A.O.R.N Dei Colli, Monaldi Hospital, Naples, Italy

**Keywords:** airway invasion, chronic obstructive pulmonary disease, machine learning–assisted risk assessment, oropharyngeal dysphagia, post-acute respiratory care, silent aspiration, videofluoroscopic swallowing study

## Abstract

**Objectives:**

To characterize swallowing impairment and aspiration risk in patients recovering from acute respiratory events and to identify clinical, functional, and anatomical predictors of airway invasion with a specific focus on Chronic Obstructive Pulmonary Disease (COPD) subgroup.

**Methods:**

In this retrospective cross-sectional study, adults hospitalized for pneumonia or acute respiratory failure underwent videofluoroscopic swallowing study (VFSS) after clinical stabilization. Airway invasion was graded using the Penetration–Aspiration Scale (PAS). Predictors of aspiration (PAS ≥ 6) were explored using multivariable logistic regression with bootstrap confidence intervals and age-adjusted marginal standardization. A Random Forest model was used to assess discrimination and identify key predictors, including a dedicated analysis in the COPD subgroup.

**Results:**

A total of 101 patients were included [mean age 60 years (range 17–85); 58.4% male]. Among them, 25 exhibited aspiration and 20% penetration despite clinical stabilization; nearly half of COPD patients fell within PAS 6–8. Vallecular and hypopharyngeal residue increased progressively across PAS categories, rising from 52 and 25% in PAS 1–2 to 84 and 100% in PAS 6–8, respectively, consistent with a severity-dependent pattern of impaired bolus clearance and increased vulnerability to airway invasion. Age-adjusted aspiration risk varied across pathologies, with the highest probabilities observed in neuromuscular diseases. The Random Forest model showed good discrimination (AUC = 0.90), identifying age and recent bronchitis as influential predictors. In COPD, semisolid aspiration and vallecular residue emerged as the most relevant physiologic predictors across both regression and machine-learning analyses.

**Conclusion:**

Swallowing impairment and aspiration are frequent after acute respiratory events, including in COPD. Recent bronchitis and pharyngeal residue represent clinically relevant markers of aspiration risk. Early, structured swallowing assessment may help identify vulnerable patients and guide targeted interventions to reduce aspiration-related respiratory morbidity.

## 1 Introduction

Swallowing is a complex sensorimotor function essential for airway protection, nutrition, and hydration, coordinated by brainstem networks that regulate the oral, pharyngeal, and esophageal phases of deglutition (1, 2). Disruption of this finely integrated process may result in penetration or aspiration of alimentary material, with clinically relevant respiratory consequences.

Oropharyngeal dysphagia is highly prevalent among older adults and patients with neurological and neuromuscular disorders (3, 4). Increasing attention has also been directed toward swallowing dysfunction in chronic respiratory diseases, where impaired breathing–swallow coordination and reduced airway clearance may further increase aspiration risk (5). In these populations, dysphagia represents an important but frequently overlooked contributor to respiratory morbidity.

Although definitions of aspiration pneumonia remain heterogeneous, dysphagia is consistently identified as a key underlying factor, often associated with involvement of gravity-dependent pulmonary segments on imaging (6–8). Aspiration pneumonia has been linked to worse clinical outcomes compared with non-aspiration pneumonia and represents a relevant subset of community-acquired and healthcare-associated respiratory infections (8, 9). Beyond pneumonia, swallowing impairment is associated with malnutrition, dehydration, chronic bronchial inflammation, reduced quality of life, and increased mortality (9–11).

Importantly, aspiration frequently occurs without overt clinical signs. Oropharyngeal dysphagia has been identified as a significant risk factor for pneumonia in older adults, with a substantial proportion of events occurring in the absence of recognized swallowing complaints (12). In elderly and neurological populations, dysphagia is also closely linked to frailty and nutritional vulnerability, which may modulate aspiration risk independently of chronological age (13, 14). After acute neurological insults such as stroke, dysphagia-related aspiration represents a major determinant of pneumonia and adverse outcomes (15).

Despite these well-established associations, swallowing disorders remain underrecognized in respiratory practice and are often overlooked or misattributed to recurrent bronchitis or acute lower respiratory tract infections. Swallowing impairment may exacerbate respiratory illness, increase the risk of aspiration pneumonia, and prolong hospitalization, yet systematic instrumental assessment is rarely incorporated into the management of patients admitted for acute respiratory events. Recent clinical statements have emphasized the importance of recognizing aspiration risk in respiratory populations, although implementation in routine practice remains inconsistent (16).

Although dysphagia-related pulmonary complications are well documented in older, neurological, and acutely ill populations, epidemiological data specifically addressing aspiration risk during the recovery phase after acute respiratory hospitalization remain limited (12, 15, 16). This relative lack of post-acute respiratory data contributes to uncertainty regarding which clinically stabilized patients may still harbor substantial swallowing-related risk.

An important clinical gap persists in the post-acute phase of respiratory illness, a stage that remains insufficiently characterized despite increasing awareness of dysphagia in respiratory medicine (3, 5). In routine practice, patients recovering from pneumonia or acute respiratory failure are often considered clinically stable once overt respiratory or hemodynamic instability has resolved, yet swallowing function is rarely assessed systematically at this stage (9, 16). As a result, residual or unrecognized airway invasion may remain undetected during a clinically relevant window in which aspiration-related respiratory morbidity may persist or recur, including recurrent infections and delayed clinical recovery (3, 8, 12).

Importantly, much of the existing literature has focused on neurological dysphagia, broadly defined aspiration pneumonia, or stable chronic respiratory disease (3, 4, 15), whereas comparatively fewer studies have specifically investigated swallowing function in patients recovering from acute respiratory decompensation and evaluated after clinical stabilization using instrumental assessments (17–20). This limits the characterization of airway invasion patterns and their clinical correlates in a population that may remain at risk despite apparent respiratory recovery.

Respiratory disease may contribute to dysphagia and airway invasion through several interacting mechanisms. These include disruption of the normal exhale–swallow–exhale sequence, reduced temporal coordination between respiration and pharyngeal swallowing, impaired cough effectiveness, decreased clearance of secretions or aspirated material, and reduced respiratory reserve during deglutition (2–5, 16). In patients recovering from acute respiratory events, these abnormalities may be further amplified by recent infection, fatigue, deconditioning, or transient post-illness weakness, thereby increasing vulnerability to inefficient bolus clearance and airway invasion.

Within this framework, COPD warrants particular attention because it combines high prevalence in respiratory practice with a pathophysiological substrate linked to impaired swallowing physiology, including alterations in breathing–swallow coordination and airway protection mechanisms (5).

Videofluoroscopic swallowing study (VFSS) was selected as the primary assessment modality because it permits simultaneous characterization of swallow timing, bolus flow, pharyngeal residue, and penetration–aspiration events across different consistencies, thereby providing a physiologically integrated evaluation of swallowing safety and efficiency (17–20). This was particularly relevant to our objectives, which extended beyond binary aspiration detection to include the identification of anatomical and functional correlates of airway invasion.

In this context, the primary objectives of this study were to characterize swallowing impairment and aspiration severity in clinically stabilized patients hospitalized for acute respiratory events and to examine clinical, functional, and anatomical correlates of airway invasion within the overall cohort. Given the clinical relevance of COPD in respiratory practice, a dedicated exploratory analysis in this subgroup was performed. Secondary analyses, including machine learning–based variable prioritization and dedicated modeling in the COPD subgroup, were explicitly conceived as exploratory and hypothesis-generating rather than confirmatory. By integrating videofluoroscopic swallowing assessment with conventional statistical modeling and machine-learning approaches, we aimed to generate clinically relevant insights applicable to routine pneumological practice, while maintaining a clear distinction between confirmatory and exploratory analytical components.

## 2 Materials and methods

### 2.1 Population and study design

We conducted a cross-sectional retrospective study of consecutive adult patients (≥ 18 years) admitted between January 2023 and 2025 to the Unit of Respiratory Pathophysiology, Monaldi-Cotugno Hospital, Naples, Italy, for acute respiratory events, including pneumonia or acute respiratory failure. All procedures were performed in accordance with the Declaration of Helsinki and applicable national regulations. A specific ethics approval number was not issued, as approval was granted through protocol identification (DISF-AR 1) for this retrospective observational study.

Patients were evaluated after clinical stabilization, defined as the resolution of acute respiratory and hemodynamic instability, as documented in the clinical records at the time of referral for VFSS. This determination was made by the treating clinical team according to routine clinical practice, in line with standard indications for safe instrumental swallowing assessment (17–20). All patients were screened after stabilization of the acute episode to ensure testing safety and feasibility. Inclusion required clinical stability, ability to cooperate and follow basic commands, and availability of essential data (age, primary diagnosis, bronchitis history).

Exclusion criteria included pediatric patients (< 18 years); individuals unable to safely undergo the test (deep sedation, coma, severe agitation, or major cognitive impairment); patients with acute hemodynamic or respiratory instability or specific contraindications to instrumental evaluation (e.g., active epistaxis, recent skull-base surgery); and those with missing essential information (bronchitis history or diagnostic classification).

Variables included age, diagnosis, bronchitis history within 3 or 6 months, and frequency of respiratory infections. Each eligible patient underwent an instrumental swallowing assessment.

### 2.2 Swallowing assessment

Swallowing physiology was evaluated using a Video-fluoroscopic Swallowing Study (VFSS), systematically recording functional findings across predefined swallowing components and relevant anatomical levels (oral preparation, swallow initiation, airway invasion, and pharyngeal residue) (17–20). The examination followed standardized clinical VFSS procedures consistent with established swallowing assessment frameworks (17–20), employing lateral-plane imaging and administering liquid, semisolid, and solid boluses. Semisolid consistency refers to an intermediate bolus texture (e.g., puree- or pudding-like) as commonly used in conventional VFSS protocols. Observations included oral preparation (bolus preparation and control), swallow initiation (timing and triggering of the pharyngeal phase), oropharyngeal airway invasion (evidence of penetration or aspiration events), vallecular residue (pooling in the valleculae), hypopharyngeal residue (stasis at the level of the pyriform sinuses), and pharyngo-esophageal involvement (upper esophageal sphincter opening and coordination). Severity was graded using the Penetration–Aspiration Scale (PAS; 1–8), an eight-point ordinal scale describing the depth of airway invasion and the patient’s ability to clear the material from the airway. Dysphagia categories were defined as normal airway protection (PAS ≤ 2), penetration (PAS 3–5), and aspiration (PAS 6–8).

## 3 Statistical and machine learning analysis

Descriptive analyses were used to characterize the cohort. Categorical variables were reported as frequencies and percentages, with exact binomial 95% confidence intervals for key prevalence estimates. Continuous variables were summarized as means ± SD or medians with interquartile ranges, as appropriate. Group comparisons were performed using chi-square or Fisher’s exact tests for categorical variables and *t*-tests or ANOVA for continuous measures.

Multivariable logistic regression and Random Forest analyses were used as complementary approaches. Logistic regression was applied to estimate adjusted associations between predictors and aspiration, whereas Random Forest modeling was used to evaluate discriminative performance and to rank variables according to their contribution to classification, accounting for potential non-linear relationships and interactions.

Predictors of aspiration (PAS ≥ 6) were evaluated using L2-regularized logistic regression (liblinear solver). Continuous predictors were median-imputed and standardized; results were expressed as odds ratios with 95% bootstrap confidence intervals (500 resamples). Differences in PAS distribution across diagnostic groups were assessed using a chi-square test applied to a 2 × 6 table after PAS dichotomization. Age-adjusted aspiration probabilities were derived from logistic regression followed by marginal standardization with bootstrap 95% confidence intervals.

A Random Forest (RF) classifier (500 trees) was implemented to predict aspiration (PAS ≥ 6), using median-imputed and standardized continuous variables and one-hot encoded categorical features. Class imbalance was addressed using inverse-frequency class weights. The dataset was partitioned using a stratified 80/20 train–test split to preserve class distribution, and a fixed random state was applied to ensure analytical stability. Internal cross-validation procedures were employed during model development to assess robustness and mitigate overfitting risk.

Model performance was quantified by Area Under the Receiver Operating Characteristic Curve (AUC) but also calibration. Calibration was assessed using the Brier score and visually examined through calibration plots; where appropriate, calibration intercept and slope were also considered to evaluate agreement between predicted and observed aspiration risk. A COPD-specific RF model (500 trees, balanced class weighting) was constructed to explore physiologically relevant predictors within this subgroup. Binary predictors included clinically relevant variables coded as present/absent (1/0), such as recent bronchitis, vallecular residue, hypopharyngeal residue, and aspiration according to bolus consistency, as well as sex. These variables were used as candidate predictors of aspiration, defined as PAS ≥ 6.

To assess the robustness of findings within the COPD subgroup, additional internal validation analyses were performed, including bootstrap resampling, repeated Random Forest modeling, and evaluation of variable-ranking consistency across modeling approaches. In the COPD subgroup, bootstrap resampling (2,000 iterations) was performed to assess the stability of regression coefficients. For each iteration, the multivariable logistic model was refitted, and the direction and magnitude of regression coefficients were recorded. Stability was evaluated in terms of consistency of coefficient direction across resampled datasets, distribution of bootstrap-derived odds ratios, and percentile-based confidence intervals.

To assess the robustness of machine learning–derived predictors, Random Forest models were repeated across multiple runs using different random seeds (200 runs). For each run, feature importance values were computed and recorded. Feature stability was evaluated based on mean feature importance, variability across runs (standard deviation), and ranking consistency, including the frequency with which each variable appeared among the top predictors.

To further evaluate the consistency of predictor selection across modeling strategies, the full ranking of variables was examined. This analysis assessed whether key predictors maintained stable relative importance across both regression-based and machine learning approaches. Finally, results from bootstrap regression and Random Forest analyses were integrated to identify predictors showing convergence across analytical frameworks. This integrated assessment was used to distinguish stable and reproducible signals from model-specific findings.

Artificial intelligence–assisted tools (ChatGPT, OpenAI) were used to generate and execute Python code implementing the machine learning analyses based on the analytical specifications provided by the authors and the uploaded dataset. The resulting outputs, including model performance metrics and feature importance rankings, were critically reviewed by the authors to ensure consistency with the dataset structure and clinical plausibility. Statistical analyses were independently reproduced and verified using GraphPad Prism version 9.1.0 (GraphPad Software, San Diego, CA, United States).

## 4 Results

### 4.1 Patient characteristics

A total of 101 patients were evaluated following hospitalization for an acute respiratory event. The mean age was 60 years (range 17–85), with a slight male predominance (59 men, 58.4%; 42 women, 41.6%). The clinical profile was heterogeneous, with the most frequent diagnosis being Amyotrophic Lateral Sclerosis (ALS) (38 patients, 37.6%), followed by Chronic Obstructive Pulmonary Disease (COPD) (28, 27.7%), other neuromuscular disorders (22, 21.8%), including non-ALS neuromuscular conditions such as myopathies and muscular dystrophies distinct from Duchenne muscular dystrophy, Duchenne Muscular Dystrophy (DMD) (6, 5.9%), fibrotic lung disease (4, 4.0%), and prior laryngectomy (3, 3.0%).

### 4.2 Swallowing severity, PAS distribution and anatomical findings across PAS categories

Swallowing performance varied widely. Over half of the patients demonstrated normal airway protection (PAS ≤ 2, 55.4%), but penetration was observed in 20 cases (19.8%), and aspiration in 25 (24.8%), indicating a substantial burden of airway invasion in this population (Table 1).

**TABLE 1:** Baseline demographic, clinical, and swallowing characteristics of patients undergoing instrumental evaluation after acute respiratory illness.

Variable	Value
Patients, n	101
Age, mean (range), years	60.3 (17–85)
Gender, n (%)	Male: 59 (58.4%); Female: 42 (41.6%)
Diagnosis, n (%)	COPD: 28 (27.7%) ALS: 38 (37.6%) DMD: 6 (5.9%) Fibrosis: 4 (4.0%) Prior laryngectomy: 3 (3.0%) Other NMD: 22 (21.8%)
Bronchitis ≤ 3 months, n (%)	52 (51.5%)
Bronchitis ≤ 6 months, n (%)	48 (47.5%)
Swallowing severity (PAS)	PAS ≤ 2 (Normal/Mild): 56 (55.4%) PAS 3–5 (Penetration): 20 (19.8%) PAS 6–8 (Aspiration): 25 (24.8%)

Swallowing severity was classified by the Penetration–Aspiration Scale (PAS). ALS, amyotrophic lateral sclerosis; DMD, Duchenne muscular dystrophy; NMD, neuromuscular disease; PAS, Penetration–Aspiration Scale. Dysphagia categories and prevalence.

Using the predefined PAS categorization, scores of 1–2 were interpreted as reflecting preserved airway protection, scores of 3–5 as laryngeal penetration without aspiration, and scores of 6–8 as aspiration. Within this framework, the distribution of PAS categories revealed not only the overall burden of aspiration, but also a substantial intermediate group with penetration, supporting a graded rather than dichotomous view of swallowing-related airway risk and highlighting a clinically relevant continuum between preserved airway protection and overt aspiration.

When stratified by diagnostic category, relevant differences in swallowing severity and airway invasion patterns emerged. To facilitate structured comparison across diagnostic subgroups, key quantitative descriptors—including PAS severity distribution and aspiration prevalence with corresponding confidence intervals—are summarized in Table 2. These data highlight clinically meaningful heterogeneity across pathologies, with respiratory and neuromuscular conditions showing distinct profiles of airway invasion severity.

**TABLE 2:** Quantitative comparison of swallowing severity and aspiration prevalence across diagnostic subgroups.

Diagnostic subgroup	N	Age (years, mean ± SD)	Male (%)	PAS 1–2 (%)	PAS 3–5 (%)	PAS 6–8 (%)	Aspiration prevalence (%)	95% CI
Respiratory disease	42	68.4 ± 11.2	61.9	45.2	23.8	31.0	31.0	18.7–45.1
Neurological disease	28	72.1 ± 9.5	53.6	39.3	28.6	32.1	32.1	16.1–52.4
Neuromuscular disease	17	64.7 ± 13.8	58.8	29.4	29.4	41.2	41.2	18.4–67.1
Other conditions	14	66.9 ± 10.6	50.0	64.3	14.3	21.4	21.4	4.7–50.8

Values are presented as mean ± standard deviation or percentages, as appropriate. PAS categories were grouped as follows: PAS 1–2 (within normal limits), PAS 3–5 (penetration without aspiration), and PAS 6–8 (aspiration). Aspiration prevalence corresponds to PAS ≥ 6. Confidence intervals (95% CI) were calculated using exact binomial methods. This table provides a structured comparison of swallowing impairment and airway invasion severity across diagnostic categories.

Examination of anatomical findings by PAS category revealed clear patterns across swallowing compartments. Vallecular residue and hypopharyngeal stasis were particularly frequent in patients with penetration and aspiration, reaching 80 and 100%, respectively, in PAS 3–5 and 84 and 100% in PAS 6–8. In contrast, preparation and initiation deficits were relatively uncommon in cases with preserved airway protection (11 and 4% in PAS 1–2) but became more prevalent with increasing PAS severity (30 and 10% in PAS 3–5; 36 and 28% in PAS 6–8).

Functional and anatomical findings influenced outcomes. Vallecular residue and post-deglutitive airway invasion were frequent among those with higher PAS scores, indicating impaired clearance and increased airway vulnerability. Older age also correlated with more severe PAS categories, consistent with known age-related changes in swallowing physiology (Table 3).

**TABLE 3:** Compartmental impairments by PAS category.

PAS category →	Preparation	Swallow initiation	Oropharynx	Valleculae	Hypopharynx	Pharyngo-esophageal
PAS 1–2	11%	4%	2%	52%	25%	5%
PAS 3–5	30%	10%	15%	80%	100%	0%
PAS 6–8	36%	28%	48%	84%	100%	4%

Values indicate the percentage of patients with findings in each compartment. For oropharynx, scores 2–3 were considered positive. Vallecular residue and hypopharyngeal involvement were common in higher PAS categories, while preparation and initiation deficits were less frequent.

### 4.3 Pathology-Specific patterns of aspiration severity

Marked differences in PAS distribution were observed across diagnostic groups. COPD showed a relatively balanced pattern, with 54.2% of patients classified within PAS 6–8 and 45.8% below the aspiration threshold (PAS < 6). In contrast, neuromuscular conditions were predominantly characterized by preserved airway protection, with 81.2% of other neuromuscular disorders and 88.2% of ALS patients remaining below PAS 6. Fibrotic lung disease showed an intermediate profile, with 66.7% of patients in the aspiration range and 33.3% below threshold. Duchenne muscular dystrophy was uniformly associated with aspiration (100% PAS 6–8), whereas prior laryngectomy patients were entirely distributed below the aspiration threshold (100% PAS < 6). Overall, these findings highlight a heterogeneous, disease-specific distribution of airway invasion severity, with COPD occupying an intermediate position between predominantly non-aspirating neuromuscular groups and uniformly aspirating conditions such as DMD

### 4.4 Age distribution across pathologies stratified by PAS severity

Age distribution varied substantially across pathologies and PAS categories (Table 4). In COPD and ALS, higher PAS categories were more frequently observed in older patients, whereas in other neuromuscular disorders, aspiration could occur at younger ages. These findings suggest that the relationship between age and aspiration severity is disease-specific rather than uniformly age-driven.

**TABLE 4:** Age distribution across pathologies stratified by PAS severity.

Pathology	PAS < 6 (Mean ± SD)	n	PAS 6–8 (Mean ± SD)	N
COPD	72.1 ± 9.3	12	70.7 ± 17.8	10
DMD	34.5 ± 8.1	5	–	0
Fibrosis	78.4 ± 6.8	2	84.0 (single case)	1
Other NMD	54.3 ± 18.5	26	37.8 ± 19.2	6
Prior laryngectomy	–	0	70.2 ± 5.3	2
ALS	62.2 ± 13.0	29	72.6 ± 7.3	4

Mean age (± standard deviation) and sample size for each pathological group stratified by PAS category (< 6 vs. 6–8). The table highlights the heterogeneous age profiles associated with different disease etiologies and aspiration severity levels.

### 4.5 Age-adjusted aspiration risk across pathologies

**FIGURE 1:**
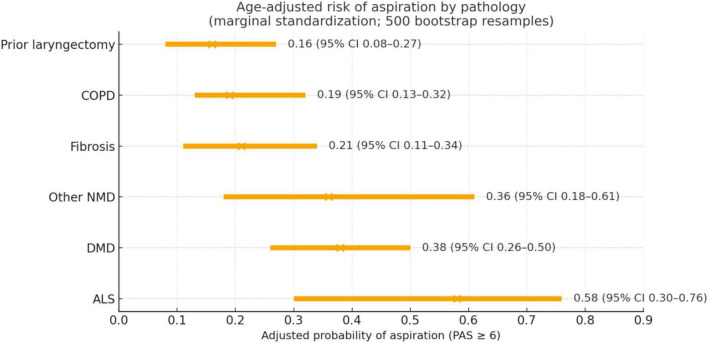
Age-adjusted probability of aspiration (PAS ≥ 6) by pathology. Points denote marginal, age-adjusted probabilities estimated from logistic regression (predictors: standardized age and pathology type); horizontal lines indicate 95% confidence intervals obtained by bootstrap (500 resamples).

Age-adjusted probabilities of aspiration varied across pathologies (Figure 1). Neuromuscular conditions showed the highest estimated risk, whereas COPD and fibrotic lung disease were associated with lower probabilities.

These results indicate that underlying pathology contributes to aspiration risk beyond the effect of age alone.

### 4.6 Machine learning analysis

**FIGURE 2:**
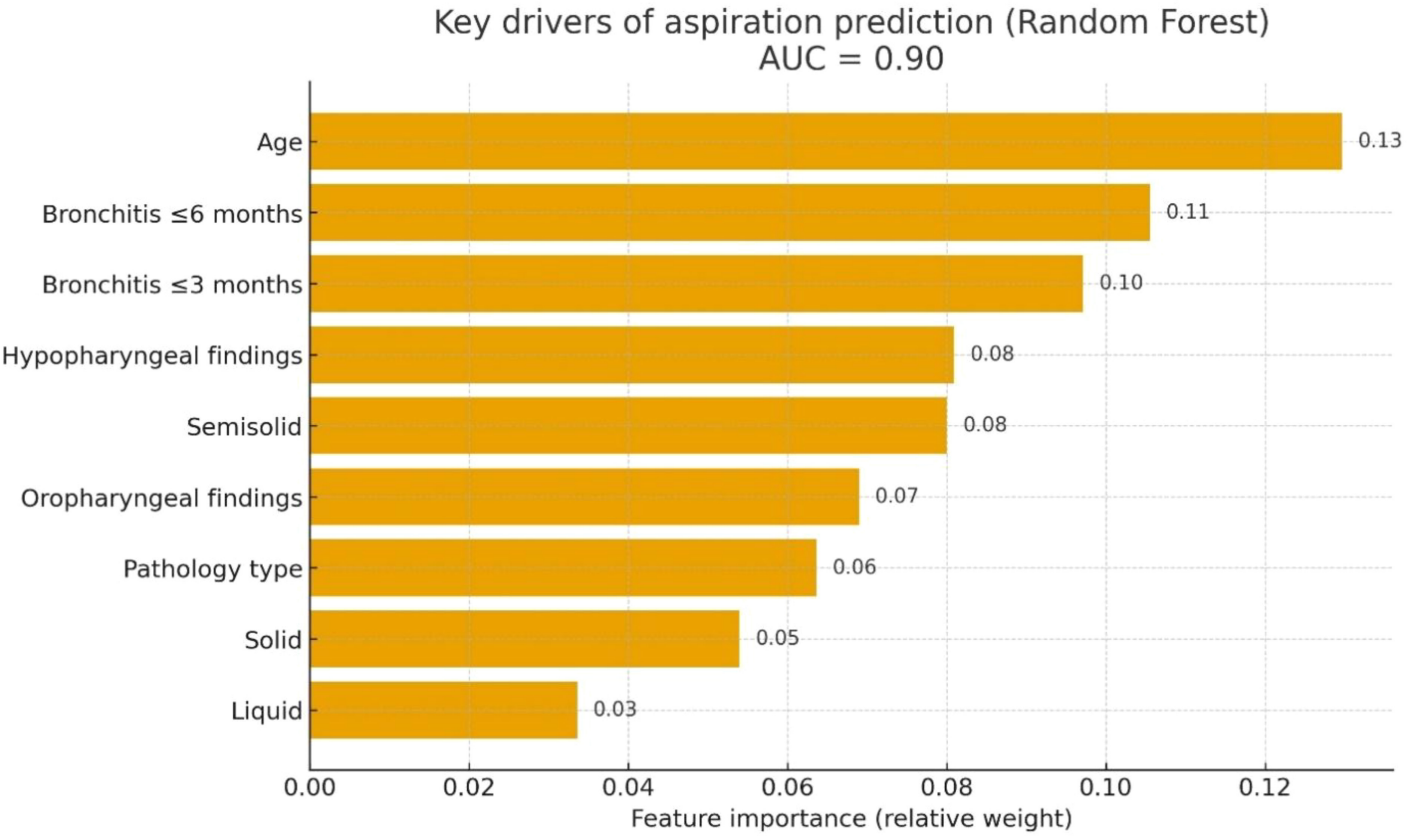
Key drivers of aspiration prediction (RF). Feature importance plot from a random forest classifier predicting aspiration (PAS ≥ 6). The model achieved an AUC of 0.90, indicating good discriminative ability. Age, Bronchitis ≤ 6 months, Bronchitis ≤ 3 months, Hypopharyngeal findings, Semisolid, Oropharyngeal findings, Pathology type, Solid, and Liquid. PAS, Penetration–Aspiration Scale. RF, Random Forest.

Machine learning (ML) provided complementary insights. On the stratified test set, the RF model achieved AUC = 0.90, indicating good discrimination for aspiration. The most influential predictors were age, bronchitis ≤ 6 months, and bronchitis ≤ 3 months, followed by hypopharyngeal findings, semisolid consistency, oropharyngeal findings, pathology type, solid, and liquid consistency (Figure 2).

### 4.7 Logistic regression for aspiration and machine learning analysis in the COPD subgroup

Twenty-three COPD patients had complete data for logistic regression. Semisolid aspiration and vallecular residue were entered as predictors based on statistical feasibility and clinical relevance. In the multivariable model, semisolid aspiration showed the strongest association with aspiration (PAS ≥ 6), although it did not reach statistical significance (adjusted OR 9.97; 95% CI 0.87–113.57; *p* = 0.064). Vallecular residue demonstrated a positive but non-significant relationship with aspiration (adjusted OR 3.87; 95% CI 0.29–51.73; *p* = 0.306). The wide confidence intervals reflect the small sample size and limited number of events, and therefore the results should be interpreted as exploratory.

These two predictors were selected because they were the only variables that met both criteria of adequate variability and acceptable collinearity within the COPD subgroup. As demonstrated in Supplementary Figures 1–3, many physiologic parameters—such as hypopharyngeal residue, impaired swallow initiation, and oropharyngeal airway invasion—showed strong intercorrelations and substantial overlap with PAS severity. Including these highly collinear variables in a small dataset would have compromised model convergence and produced unstable or non-estimable odds ratios. Semisolid aspiration and vallecular residue instead represent distinct and clinically meaningful indicators of impaired bolus propulsion and ineffective airway protection, making them the most appropriate predictors to retain in a parsimonious model.

Given the limited number of aspiration events in the COPD subgroup, the multivariable model was intentionally restricted to a parsimonious set of predictors selected based on clinical relevance, sufficient variability, and acceptable collinearity structure.

Additional robustness analyses were performed to assess whether the identified signals were stable across internal resampling procedures. In the parsimonious COPD logistic model, bootstrap resampling (2,000 iterations) demonstrated consistent directionality for both semisolid aspiration and vallecular residue, with positive coefficients observed in 98.8 and 85.4% of bootstrap samples, respectively. The corresponding median bootstrap odds ratios were 2.50 (2.5th–97.5th percentile, 1.13–5.20) for semisolid aspiration and 1.49 (2.5th–97.5th percentile, 0.70–3.26) for vallecular residue, supporting directional stability despite wide uncertainty intervals (Supplementary Table 1A).

Feature-importance stability was also examined across 200 repeated Random Forest runs restricted to the COPD subgroup. Semisolid aspiration remained the most stable signal (mean importance 0.297 ± 0.107; top-3 rank frequency 91.5%), followed by vallecular residue (0.187 ± 0.094; top-3 rank frequency 61.0%). Hypopharyngeal residue, male sex, and thin-liquid aspiration showed intermediate stability, whereas solid aspiration ranked lower overall. These analyses indicate that the prioritization of semisolid aspiration and residue-related variables was not driven by a single model fit and was directionally robust across internal resampling (Supplementary Table 1B and Supplementary Figure 4).

To further explore the consistency of variable selection across modeling strategies, we examined the full ranking of predictors and their relative importance profiles. This analysis confirmed that semisolid aspiration and residue-related variables consistently occupied top-ranking positions across models, whereas other variables showed greater variability in their relative contribution (Supplementary Table 1C).

Regression-based and machine learning–derived results were considered complementary, with regression modeling providing adjusted estimates of association and Random Forest analysis capturing broader variable importance within a non-linear feature space.

When integrating regression-based stability and machine learning–derived feature importance, semisolid aspiration and vallecular residue emerged as convergent signals across analytical approaches, supporting their relevance despite the exploratory nature of subgroup analyses (Supplementary Table 1D).

**FIGURE 3:**
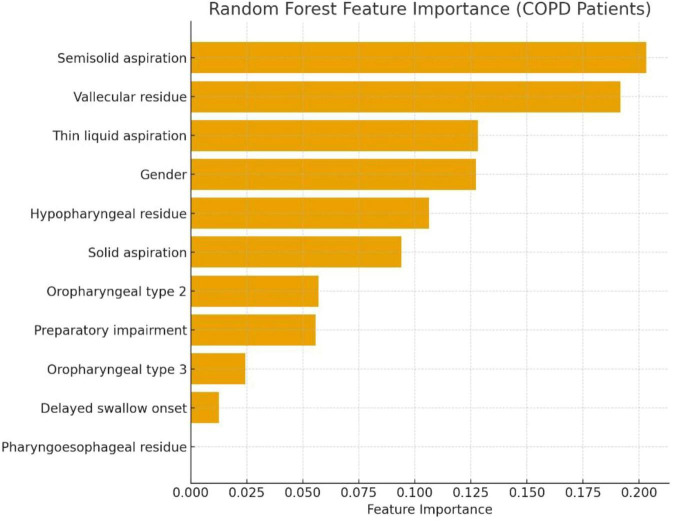
Feature importance of the Random Forest model predicting aspiration (PAS ≥ 6) in COPD patients. Horizontal bars represent the relative contribution of each videofluoroscopic or clinical variable to the RF classifier trained on the COPD subgroup Semisolid aspiration and vallecular residue were the strongest predictors of aspiration, followed by thin-liquid aspiration, sex, and hypopharyngeal residue. Variables with minimal representation (e.g., pharyngoesophageal residue) showed negligible predictive value. Importance values are based on the mean decrease in impurity across 500 trees. PAS, Penetration–Aspiration Scale. RF, Random Forest.

A RF model was trained to identify the most relevant predictors of aspiration (PAS ≥ 6) in COPD patients. The model included all VFSS and clinical variables with sufficient variability. Feature importance analysis indicated that semisolid aspiration was the strongest predictor, followed by vallecular residue and thin-liquid aspiration. Hypopharyngeal residue, solid aspiration, and sex also contributed, whereas variables with very limited representation (such as pharyngoesophageal residue or delayed swallow onset) had negligible importance. This ranking reflects the correlation structure of the dataset as documented in Supplementary Figures 1, 2 show that residue and airway-invasion measures form a closely correlated cluster, while Supplementary Figure 3 demonstrates that clinical variables such as bronchitis timing have distinct correlation patterns. These correlation profiles help explain why variables related to residue and aspiration severity produced the largest impurity reductions in the RF model. The full set of feature importance values is shown in Figure 3.

In addition to discrimination, calibration analysis was performed to assess the agreement between predicted and observed aspiration probabilities. Calibration metrics are summarized in Supplementary Table 2, and visual assessment of calibration is provided in Supplementary Figure 4. This evaluation provides complementary information on the numerical reliability of model predictions beyond rank-order classification.

## 5 Discussion

In this cohort of patients evaluated after hospitalization for acute respiratory events, swallowing impairment and airway invasion were frequent despite clinical stabilization, with approximately one quarter of patients exhibiting aspiration on videofluoroscopic swallowing study (VFSS). These findings identify dysphagia as a common and potentially modifiable contributor to respiratory morbidity in acute respiratory inpatients. Vallecular and hypopharyngeal residue consistently emerged as anatomical correlates of airway invasion, indicating impaired bolus clearance as a central pathophysiological feature of aspiration vulnerability.

Within this overall pattern, aspiration risk varied substantially across underlying conditions. Neuromuscular diseases demonstrated the highest age-adjusted probabilities of aspiration, consistent with progressive bulbar and pharyngeal muscle involvement and prior evidence linking swallowing dysfunction, nutritional impairment, and disease severity in neurodegenerative disorders (14, 21–23). COPD constituted one of the largest clinically relevant strata, with nearly half of patients exhibiting severe airway invasion (PAS 6–8). Together, these findings indicate that dysphagia-related aspiration remains underrecognized in routine pneumological practice, as objective airway invasion was frequently identified in the absence of overt swallowing complaints (24–26).

Pharyngeal residue, particularly in the vallecular and hypopharyngeal compartments, is physiologically relevant not merely as a correlate of dysphagia severity, but as an indicator of impaired bolus propulsion and incomplete pharyngeal clearance. From a biomechanical perspective, residue reflects reduced pharyngeal constriction, impaired timing and coordination of swallowing events, and inefficient clearance of ingested material. Under these conditions, residual material may subsequently enter the airway during post-swallow respiration, secondary swallows, or delayed spillover, particularly when laryngeal closure, sensory feedback, or cough-mediated clearance are compromised (16–20, 27). In this sense, residue can be understood as an intermediate biomechanical phenotype linking inefficient swallowing to subsequent airway invasion, rather than a purely static descriptor of swallowing impairment.

Silent aspiration—airway invasion occurring without protective coughing—is a recognized contributor to dysphagia-related pneumonia and aspiration-related lung injury (15, 17, 28–30). Effective airway protection depends on coordinated mechanisms, including glottal closure and an intact cough reflex; impairment of these defenses in neurological disease, altered mental status, esophageal motility disorders, age-related swallowing changes, and post-surgical or post-intubation states increase susceptibility to aspiration (31–35). In this context, recent or recurrent bronchitis should be interpreted as a clinical correlate potentially reflecting impaired airway clearance or increased vulnerability to aspiration, rather than as a direct marker of silent aspiration, which was not specifically assessed in this study.

Consistent with this framework, a recent history of bronchitis emerged as a salient clinical correlate of aspiration in the present cohort. Deficits in oral preparation were identified as independent determinants of aspiration, underscoring the contribution of impaired bolus formation and control to downstream pharyngeal inefficiency and compromised airway protection.

Across PAS severity classes, vallecular residue and hypopharyngeal stasis were highly prevalent among individuals with penetration and aspiration, while deficits in bolus preparation and swallow initiation increased progressively with higher PAS scores. Together, these findings delineate a severity-dependent continuum of swallowing dysfunction, in which cumulative inefficiencies across swallowing phases translate into escalating airway invasion.

Post-swallow vallecular residue is widely recognized as a marker of swallowing inefficiency (36–38). Although residue does not inevitably result in aspiration, it can act as a reservoir for subsequent airway invasion, particularly in the absence of effective clearing mechanisms (37, 38). Its clinical impact is modulated by bolus characteristics and clearance capacity (37). Accordingly, significant vallecular residue on instrumental assessment should be regarded as a clinically meaningful risk feature and may guide compensatory strategies and rehabilitative interventions (38).

Age-related trends were associated with increasing dysphagia severity, but their clinical significance differed across disease groups. In COPD and ALS, higher PAS scores clustered predominantly in older patients, consistent with reduced airway protective capacity. In contrast, other neuromuscular disorders showed a divergent pattern, with younger patients more frequently reaching PAS 6–8, suggesting that disease progression rather than chronological aging may predominate in determining aspiration vulnerability.

Given the high prevalence of COPD in respiratory practice, even a moderate-to-high aspiration burden may translate into substantial population-level consequences (39, 40). These findings support incorporating targeted swallowing assessment in COPD alongside traditionally recognized high-risk neuromuscular and post-laryngectomy populations, in line with respiratory guidance emphasizing early identification of aspiration risk (41, 42).

Building on these clinical observations, machine-learning analyses complemented conventional regression by prioritizing variables according to their contribution to classification performance. The multivariable regression and Random Forest analyses were interpreted as complementary rather than interchangeable, with regression modeling providing adjusted estimates of association and Random Forest analysis assessing discriminative performance and ranking variables according to their contribution within a potentially non-linear feature space. The model demonstrated good discriminative performance and consistently highlighted age and bronchitis history among the most influential predictors (24, 25, 43). This interpretation is further supported by physiological evidence showing that impaired coordination between breathing and swallowing in COPD may compromise airway protection and increase susceptibility to airway invasion (44, 45). Within the COPD subgroup, semisolid aspiration and vallecular residue emerged as relevant contributors across analytic approaches, despite not fulfilling strict criteria for statistical independence. This divergence reflects methodological constraints rather than conflicting biological signals and should be considered hypothesis-generating. Importantly, variable importance in the machine learning model should not be interpreted as evidence of independent causal effects.

The robustness of these findings within the COPD subgroup was further supported by complementary analytical approaches. Bootstrap-based regression analyses demonstrated high directional stability for semisolid aspiration and moderate stability for vallecular residue (Supplementary Table 1A), while repeated Random Forest analyses confirmed stable prioritization of these variables (Supplementary Table 1B and Supplementary Figure 4). Examination of full variable-ranking profiles further supported the consistent importance of these predictors (Supplementary Table 1C). Importantly, convergence across modeling frameworks suggests that these findings reflect a reproducible signal rather than a model-specific artifact (Supplementary Table 1D).

Concordance between regression-based associations and machine learning–derived variable importance was considered supportive when similar physiologic or clinical domains emerged across methods, but differences were expected because the two approaches address distinct analytical questions. In particular, variable importance in Random Forest models reflects contribution to classification performance within the full feature space rather than adjusted independence in the regression sense.

From a physiological perspective, this consistency reinforces the interpretation of semisolid aspiration and pharyngeal residue as clinically meaningful markers of impaired bolus clearance and airway protection in COPD, rather than artifacts of model specification. Nevertheless, these internal checks do not substitute for external validation, and the subgroup findings should still be interpreted as exploratory. Given the limited subgroup size, these results should be considered hypothesis-generating and warrant confirmation in larger, prospectively designed cohorts

Our findings are consistent with the broader literature indicating that swallowing dysfunction may persist beyond the phase of overt respiratory instability and may remain clinically relevant during recovery from acute illness. While dysphagia has been extensively characterized in neurological populations and in post-extubation settings, comparatively fewer studies have focused on patients recovering from acute respiratory events outside the critical care context. Evidence from chronic respiratory disease, aspiration pneumonia, and critical care populations supports the plausibility of persistent airway-protection deficits after apparent clinical stabilization, including impaired breathing–swallow coordination, reduced airway clearance, and increased susceptibility to aspiration-related complications (5, 15, 16, 28–34, 39, 40). In this context, our findings extend the existing literature by providing systematic instrumental characterization of swallowing function in clinically stabilized respiratory inpatients, highlighting a phase of vulnerability that may not be captured by routine respiratory assessment alone.

Taken together, these results support the systematic integration of respiratory history with instrumental swallowing assessment in patients hospitalized for acute respiratory events, particularly those with recent or recurrent bronchitis and neuromuscular disease.

From a clinical implementation perspective, these findings do not support universal instrumental assessment in all patients recovering from acute respiratory events. Rather, they support a targeted, risk-based strategy aligned with routine respiratory care workflows. In this framework, patients with recent bronchitis, recurrent lower respiratory infections, neuromuscular disease, COPD with suggestive symptoms or high-risk physiological profiles, and objective signs of impaired secretion handling or nutritional vulnerability may represent priority candidates for formal swallowing evaluation.

A stepwise approach may be particularly feasible in clinical practice. Bedside swallowing screening can serve as an initial triage tool to identify patients at higher risk, while videofluoroscopic swallowing study (VFSS) may be reserved for cases in which detailed assessment of residue, swallow timing, and airway invasion is likely to influence clinical decision-making. This approach allows efficient allocation of resources while maintaining clinical sensitivity to relevant swallowing impairment.

Importantly, implementation of this strategy is facilitated by integration within multidisciplinary care pathways, including collaboration between respiratory physicians, speech and swallowing specialists, and rehabilitation teams. Such coordination is particularly feasible in tertiary or high-complexity settings and may improve identification of patients at risk of aspiration-related complications during the post-acute phase.

### 5.1 Strengths and limitations

This study presents several noteworthy strengths. It systematically integrates instrumental swallowing assessment into the evaluation of patients hospitalized for acute respiratory events—a population in whom dysphagia is frequently underrecognized despite its potential respiratory consequences. Unlike prior investigations focused predominantly on neurological cohorts or post-stroke aspiration, this work examines swallowing physiology within a broader respiratory inpatient population, including a clinically meaningful COPD subgroup. The combined use of multivariable regression and machine-learning approaches represents an additional strength, as this complementary analytical framework enables identification of statistically independent predictors while also exploring data-driven variable prioritization and potential non-linear interactions. Furthermore, the application of videofluoroscopic swallowing study (VFSS) allowed detailed compartmental characterization of residue and airway invasion patterns, providing mechanistic insights beyond binary aspiration classification. The real-world clinical setting enhances the translational relevance of the findings, as the cohort reflects routine respiratory practice rather than a highly selected experimental population.

An additional strength of the revised analysis is that internal robustness checks were performed within the COPD subgroup. Bootstrap resampling supported the directional stability of the parsimonious regression coefficients, and repeated Random Forest runs showed that semisolid aspiration and vallecular residue consistently remained among the highest-ranked predictors. Nevertheless, these internal checks do not substitute for external validation, and the subgroup findings should be interpreted as exploratory and hypothesis-generating, requiring confirmation in larger prospective cohorts.

Several limitations should be acknowledged. The retrospective cross-sectional design represents a key limitation. Because exposure variables and swallowing outcomes were assessed within an observational framework after clinical stabilization, temporal sequencing cannot be established with certainty, and causal inferences should be avoided. Accordingly, identified associations—particularly those involving recent bronchitis, residue, and aspiration—should be interpreted as clinically informative but not causally demonstrative. Furthermore, bolus consistencies were recorded according to routine clinical VFSS practice and were not classified using a standardized framework such as the International Dysphagia Diet Standardization Initiative (IDDSI). This may limit comparability with studies employing formalized consistency classifications.

Moreover, the overall cohort size was modest, and several diagnostic subgroups were small. This limited statistical power, widened confidence intervals, increased the risk of unstable estimates in subgroup models, and reduced the ability to detect moderate associations reliably. These constraints were especially relevant for the COPD subgroup analysis and for rarer categories such as fibrosis or prior laryngectomy.

Generalizability is also limited. The cohort was derived from a hospitalized population in a tertiary respiratory setting and therefore likely reflects a higher burden of clinical complexity, comorbidity, and referral bias than would be expected in outpatient or community-based populations. The findings should not be extrapolated uncritically to lower-acuity respiratory settings or to unselected general medical populations.

Furthermore, additional unmeasured confounding may have influenced the observed associations. Variables such as nutritional status, frailty severity, subtle cognitive dysfunction, medication burden (including sedating or anticholinergic agents), oral health, and detailed respiratory support history were not systematically available in the dataset. These factors may affect both swallowing physiology and respiratory outcomes and should be incorporated into future prospective studies to refine risk stratification

Although internal validation procedures were applied, the machine learning analyses should be interpreted as exploratory and hypothesis-generating. Given the limited dataset and absence of external validation, model performance estimates, including AUC values, should be considered cautiously, as potential overfitting cannot be fully excluded. In addition, while analytical outputs were critically examined by the authors for statistical consistency and clinical plausibility, independent computational replication of the complete machine learning pipeline was not performed prior to submission. Future multicenter, prospective investigations with larger cohorts and external validation are warranted to confirm and extend these findings.

### 5.2 Clinical implications and future directions

Clinically, these findings highlight the importance of integrating recent respiratory history into dysphagia assessment pathways. Patients with bronchitis or lower respiratory tract infection in the preceding months may warrant heightened surveillance for aspiration even in the absence of overt neurological disease. Early instrumental evaluation may facilitate timely intervention and reduce the risk of recurrent infection or hospitalization. Future prospective studies should validate these predictors and assess whether ML-assisted stratification improves targeted management strategies.

## 6 Conclusion

The present findings support systematic dysphagia screening following acute respiratory hospitalization, particularly in patients with recent respiratory infections or neuromuscular disease, and identify COPD as a clinically important group with a substantial and often underrecognized aspiration burden. Integrating respiratory history with instrumental swallowing assessment may help guide timely interventions and reduce aspiration-related morbidity in pneumological practice.

## Data Availability

The raw data supporting the conclusions of this article will be made available by the authors, without undue reservation.

## References

[1] Lang I. Brain stem control of the phases of swallowing. *Dysphagia.* (2009) 24:333–48. 10.1007/s00455-009-9211-6 19399555

[2] Ambrocio K Miles A Bhutada A Choi D Garand K. Defining normal sequential swallowing biomechanics. *Dysphagia.* (2023) 38:1497–510. 10.1007/s00455-023-10576-z 37097448 PMC11554329

[3] Marik P Kaplan D. Aspiration pneumonia and dysphagia in the elderly. *Chest.* (2003) 124:328–36. 10.1378/chest.124.1.328 12853541

[4] Kawashima K Motohashi Y Fujishima I. Prevalence of dysphagia among community-dwelling elderly individuals as estimated using a questionnaire for dysphagia screening. *Dysphagia.* (2004) 19:266–71. 10.1007/s00455-004-0013-6 15667063

[5] Verin E Clavé P Bonsignore M Marie J Bertolus C Similowski T et al. Oropharyngeal dysphagia: when swallowing disorders meet respiratory diseases. *Eur Respir J.* (2017) 49:1602530. 10.1183/13993003.02530-2016 28404653

[6] Simonetti A Viasus D Garcia-Vidal C Adamuz J Roset A Manresa F et al. Timing of antibiotic administration and outcomes of hospitalized patients with community-acquired and healthcare-associated pneumonia. *Clin Microbiol Infect.* (2012) 18:1149–55. 10.1111/j.1469-0691.2011.03709.x 22115052

[7] Garcia-Vidal C Viasus D Roset A Adamuz J Verdaguer R Dorca J et al. Low incidence of multidrug-resistant organisms in patients with healthcare-associated pneumonia requiring hospitalization. *Clin Microbiol Infect.* (2011) 17:1659–65. 10.1111/j.1469-0691.2011.03484.x 21463391

[8] Komiya K Ishii H Umeki K Mizunoe S Okada F Johkoh T et al. Impact of aspiration pneumonia in patients with community-acquired pneumonia and healthcare-associated pneumonia: a multicenter retrospective cohort study. *Respirology.* (2013) 18:514–21. 10.1111/resp.12029 23231701

[9] Connolly M. Of proverbs and prevention: aspiration and its consequences in older patients. *Age Ageing.* (2010) 39:2–4. 10.1093/ageing/afp214 20015854

[10] Carrión S Cabré M Monteis R Roca M Palomera E Serra-Prat M et al. Oropharyngeal dysphagia is a prevalent risk factor for malnutrition in a cohort of older patients admitted with an acute disease to a general hospital. *Clin Nutr.* (2015) 34:436–42. 10.1016/j.clnu.2014.04.014 24882372

[11] Rosenbek J Robbins J Roecker E Coyle J Wood JLA. penetration-aspiration scale. *Dysphagia.* (1996) 11:93–8. 10.1007/BF00417897 8721066

[12] Almirall J Rofes L Serra-Prat M Icart R Palomera E Arreola V et al. Oropharyngeal dysphagia is a risk factor for community-acquired pneumonia in the elderly. *Eur Respir J.* (2013) 41:923–8. 10.1183/09031936.00019012 22835620

[13] Yang R Yang A Chen Y Lee S Lee S Chen J. Association between dysphagia and frailty in older adults: a systematic review and meta-analysis. *Nutrients.* (2022) 14:1812. 10.3390/nu14091812 35565784 PMC9105461

[14] Pizzorni N Ciammola A Casazza G Ginocchio D Bianchi F Feroldi S et al. Predictors of malnutrition risk in neurodegenerative diseases: the role of swallowing function. *Eur J Neurol.* (2022) 29:2493–8. 10.1111/ene.15345 35384164 PMC9540307

[15] Martino R Foley N Bhogal S Diamant N Speechley M Teasell R. Dysphagia after stroke: incidence, diagnosis, and pulmonary complications. *Stroke.* (2005) 36:2756–63. 10.1161/01.STR.0000190056.76543.eb 16269630

[16] Simpson A Allen J Chatwin M Crawford H Elverson J Ewan V et al. BTS clinical statement on aspiration pneumonia. *Thorax.* (2023) 78:S3–21. 10.1136/thorax-2022-219699 36863772

[17] Martin-Harris B Jones B. The videofluorographic swallowing study. *Phys Med Rehabil Clin N Am.* (2008) 19:769–85. 10.1016/j.pmr.2008.06.004 18940640 PMC2586156

[18] Matsuo K Palmer J. Video fluoroscopic techniques for the study of oral food processing. *Curr Opin Food Sci.* (2016) 9:1–10. 10.1016/j.cofs.2016.03.004 27213138 PMC4871608

[19] Tomita S Oeda T Umemura A Kohsaka M Park K Yamamoto K et al. Video-fluoroscopic swallowing study scale for predicting aspiration pneumonia in Parkinson’s disease. *PLoS One.* (2018) 13:e0197608. 10.1371/journal.pone.0197608 29874285 PMC5991364

[20] Chang M Kwak S. Videofluoroscopic swallowing study findings associated with subsequent pneumonia in patients with dysphagia due to frailty. *Front Med.* (2021) 8:690968. 10.3389/fmed.2021.690968 34291064 PMC8287055

[21] Audag N Toussaint M Liistro G Vandervelde L Cugy E Reychler G. European survey: dysphagia management in patients with neuromuscular diseases. *Dysphagia.* (2022) 37:1279–87. 10.1007/s00455-021-10392-3 34977983

[22] Mamarabadi M Mauney S Li Y Aboussouan L. Evaluation and management of dyspnea as the dominant presenting feature in neuromuscular disorders. *Muscle Nerve.* (2024) 70:916–28. 10.1002/mus.28243 39267292

[23] Audag N Goubau C Toussaint M Reychler G. Screening and evaluation tools of dysphagia in adults with neuromuscular diseases: a systematic review. *Ther Adv Chronic Dis.* (2019) 10:2040622318821622. 10.1177/2040622318821622 30728931 PMC6357297

[24] Baijens L Clavé P Cras P Ekberg O Forster A Kolb G et al. European society for swallowing disorders - European union geriatric medicine society white paper: oropharyngeal dysphagia as a geriatric syndrome. *Clin Interv Aging.* (2016) 11:1403–28. 10.2147/CIA.S107750 27785002 PMC5063605

[25] Zhang Y Gong Z Cai J Yu W Dai Y Wang H. Incidence of dysphagia-related safety incidents in older adults across feeding methods: a systematic review and meta-analysis. *J Nutr Health Aging.* (2025) 29:100522. 10.1016/j.jnha.2025.100522 39985956 PMC12180055

[26] Huang P Hsu Y Li C Hsieh S Lee K Wu K et al. Videofluoroscopy dysphagia severity scale is predictive of subsequent remote pneumonia in dysphagia patients. *Int J Med Sci.* (2023) 20:429–36. 10.7150/ijms.76448 36860676 PMC9969506

[27] Borders J Brates D. Use of the penetration-aspiration scale in dysphagia research: a systematic review. *Dysphagia.* (2020) 35:583–97. 10.1007/s00455-019-10064-3 31538220

[28] Ramsey D Smithard D Kalra L. Silent aspiration: what do we know? *Dysphagia.* (2005) 20:218–25. 10.1007/s00455-005-0018-9 16362510

[29] Irwin G Leatherman J. Dysphagia. *Prim Care.* (2025) 52:171–9. 10.1016/j.pop.2024.09.016 39939087

[30] Komiya K Yoshimatsu Y Hagiwara A Kudoh R Shuto H Yamatani I et al. Pneumonia in frail older adults: from diagnosis to optimized management. *J Infect Chemother.* (2026) 32:102914. 10.1016/j.jiac.2026.102914 41580281

[31] Yu W Dan L Cai J Wang Y Wang Q Zhang Y et al. Incidence of post-extubation dysphagia among critical care patients undergoing orotracheal intubation: a systematic review and meta-analysis. *Eur J Med Res.* (2024) 29:444. 10.1186/s40001-024-02024-x 39217392 PMC11365263

[32] Troll C Trapl-Grundschober M Teuschl Y Cerrito A Compte M Siegemund MA. bedside swallowing screen for the identification of post-extubation dysphagia on the intensive care unit - validation of the Gugging swallowing screen (GUSS)-ICU. *BMC Anesthesiol.* (2023) 23:122. 10.1186/s12871-023-02072-6 37055724 PMC10099025

[33] Brodsky M Pandian V Needham D. Post-extubation dysphagia: a problem needing multidisciplinary efforts. *Intensive Care Med.* (2020) 46:93–6. 10.1007/s00134-019-05865-x 31768568 PMC7219527

[34] de Sire A Ferrillo M Lippi L et al. Sarcopenic dysphagia, malnutrition, and oral frailty in elderly: a comprehensive review. *Nutrients.* (2022) 14:982. 10.3390/nu14050982 35267957 PMC8912303

[35] Sarbinowska J Wiatrak B Waśko-Czopnik D. Esophageal motility disorders in the natural history of acid-dependent causes of dysphagia and their influence on patients’ quality of life-a prospective cohort study. *Int J Environ Res Public Health.* (2021) 18:11138. 10.3390/ijerph182111138 34769657 PMC8583542

[36] Sabry A Abou-Elsaad T. Pharyngeal residue severity and aspiration risk in stroke patient using fiber-optic endoscopic evaluation of swallowing. *Folia Phoniatr Logop.* (2023) 75:158–63. 10.1159/000528204 36412739

[37] Molfenter S Steele C. The relationship between residue and aspiration on the subsequent swallow: an application of the normalized residue ratio scale. *Dysphagia.* (2013) 28:494–500. 10.1007/s00455-013-9459-8 23460344

[38] Oliveira D Moreira E de Freitas M Gonçalves J Furkim A Clavé P. Pharyngeal residue and aspiration and the relationship with clinical/nutritional status of patients with oropharyngeal dysphagia submitted to videofluoroscopy. *J Nutr Health Aging.* (2017) 21:336–41. 10.1007/s12603-016-0754-6 28244575 PMC12879892

[39] Lin T Shune S. Chronic obstructive pulmonary disease and dysphagia: a synergistic review. *Geriatrics.* (2020) 5:45. 10.3390/geriatrics5030045 32847110 PMC7554843

[40] Cvejic L Bardin P. Swallow and aspiration in chronic obstructive pulmonary disease. *Am J Respir Crit Care Med.* (2018) 198:1122–9. 10.1164/rccm.201804-0704PP 29939762

[41] Cvejic L Harding R Churchward T Turton A Finlay P Massey D et al. Laryngeal penetration and aspiration in individuals with stable COPD. *Respirology.* (2011) 16:269–75. 10.1111/j.1440-1843.2010.01875.x 21054669

[42] Zhu T Yang M Weng L Cheng F. Risk factors for dysphagia in elderly patients with COPD: a systematic review and meta-analysis protocol. *BMJ Open.* (2025) 15:e105405. 10.1136/bmjopen-2025-105405 40973369 PMC12458662

[43] Nativ-Zeltzer N Nachalon Y Kaufman M Seeni I Bastea S Aulakh S et al. Predictors of aspiration pneumonia and mortality in patients with dysphagia. *Laryngoscope.* (2022) 132:1172–6. 10.1002/lary.29770 34313344

[44] Gross RD Atwood CW Ross SB Olszewski JW Eichhorn KA. The coordination of breathing and swallowing in chronic obstructive pulmonary disease. *Am J Respiratory Crit Care Med.* (2009) 179:559–65. 10.1164/rccm.200807-1139OC 19151193

[45] Sagi O Rokach L. Explainabledecisionforest: transforming a decision forest into an interpretable tree. *Inf Fusion.* (2020) 61:124–38. 10.1016/j.inffus.2020.03

