# Supplementary material for: Swallowing impairment and aspiration risk in clinically stabilized patients hospitalized for acute respiratory events: a cohort-wide machine-learning analysis with COPD-specific insights
Source: Front Med (Lausanne). 2026 May 5;13:1804250. doi: 10.3389/fmed.2026.1804250 (PMC13183647; doi:10.3389/fmed.2026.1804250)
Supplement: Supplementary file 1 [file Supplementary_file_1.docx]

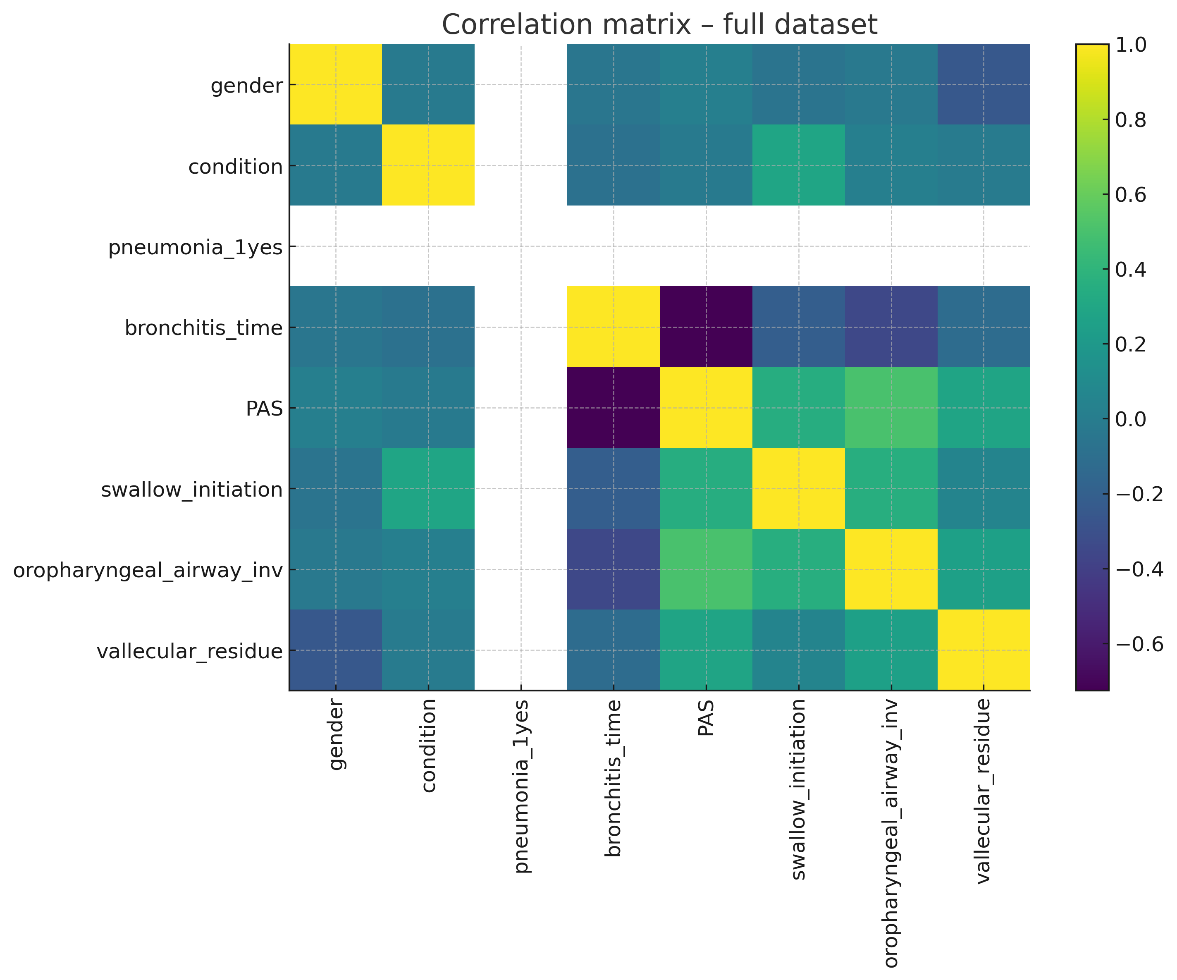


**Figure S1. Full correlation matrix of clinical and physiologic variables.**

Heatmap displaying the pairwise Pearson correlation coefficients among all numeric clinical and swallowing-related variables included in the analysis . Continuous variables (e.g. age, PAS score) and dichotomized categorical variables (e.g. pneumonia, bronchitis timing, vallecular residue, hypopharyngeal residue, oropharyngeal airway invasion) were analyzed after recoding yes/no responses into 1/0. This figure illustrates the overall correlation structure of the dataset and highlights patterns of collinearity between clinical history and pharyngeal physiologic measures.


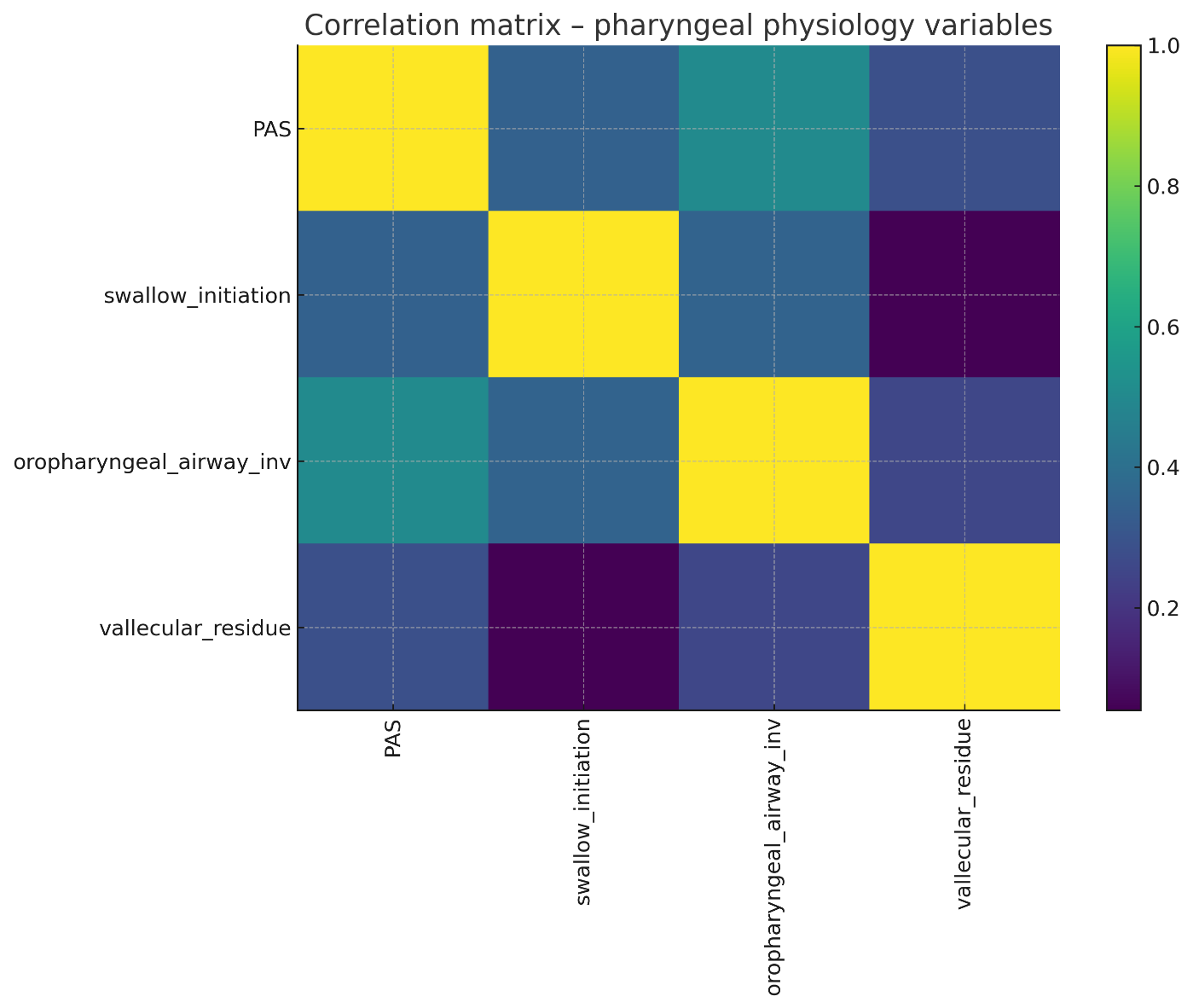


**Figure S2. Correlation matrix of pharyngeal physiology variables.** Heatmap showing Pearson correlations among key pharyngeal physiologic measures: PAS score, swallow initiation, oropharyngeal airway invasion, vallecular residue, and hypopharyngeal residue. All variables were coded numerically before analysis. This figure emphasizes the inter-relationships among biomechanical swallowing parameters and their degree of overlap in capturing pharyngeal dysfunction.


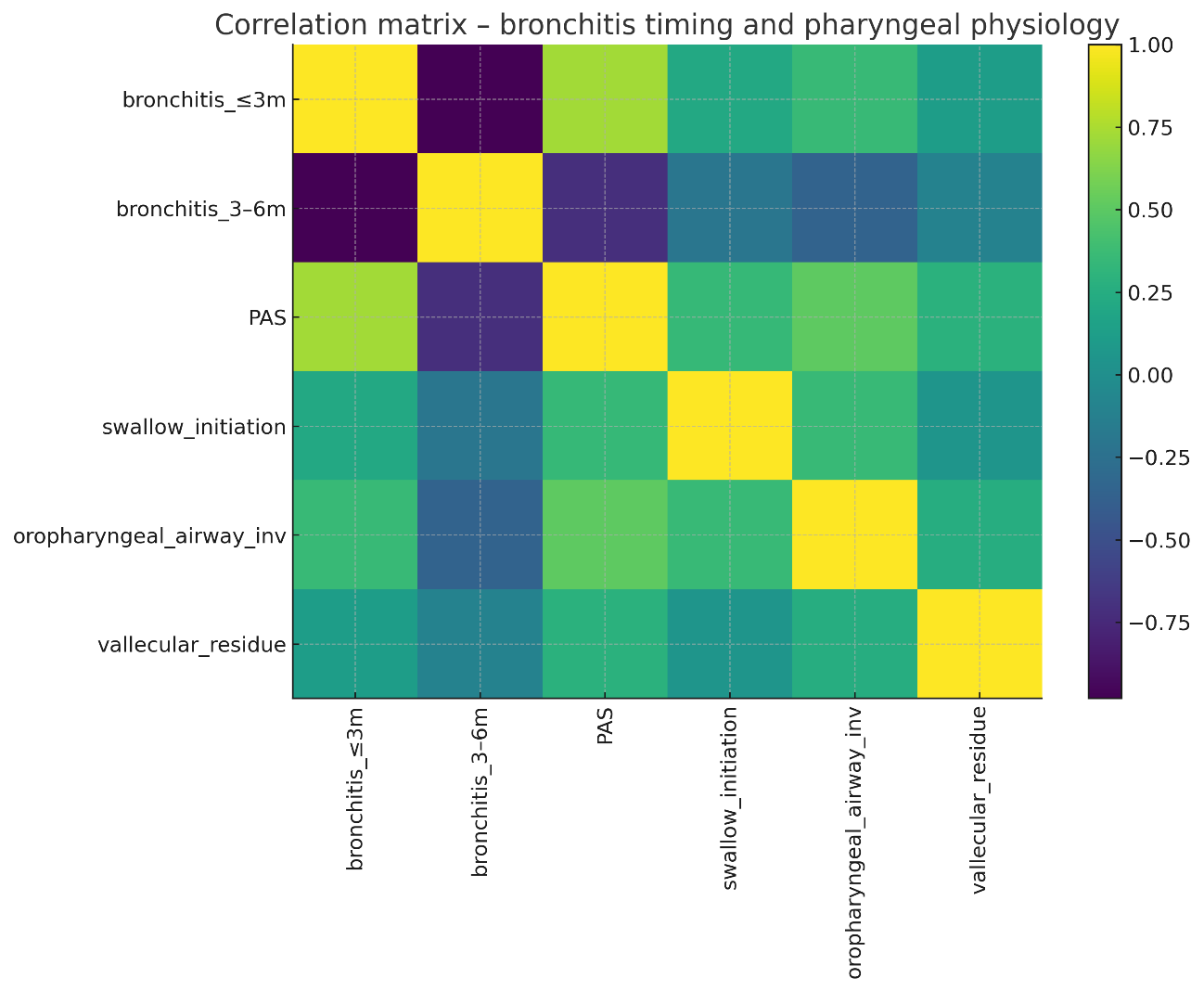


**Figure S3. Correlation matrix for bronchitis timing and pharyngeal physiology.** Heatmap illustrating Pearson correlations between bronchitis within 3 months (bronchitis_≤3m), bronchitis 3–6 months prior (bronchitis_3–6m), and pharyngeal physiologic variables (PAS score, swallow initiation, oropharyngeal airway invasion, vallecular residue, hypopharyngeal residue). Bronchitis timing was encoded as separate binary indicators for episodes ≤3 months and 3–6 months before assessment. This figure highlights the stronger collinearity of recent bronchitis with markers of severe dysfunction and the comparatively weaker correlations for bronchitis 3–6 months, supporting its more independent contribution in the Random Forest model.

**
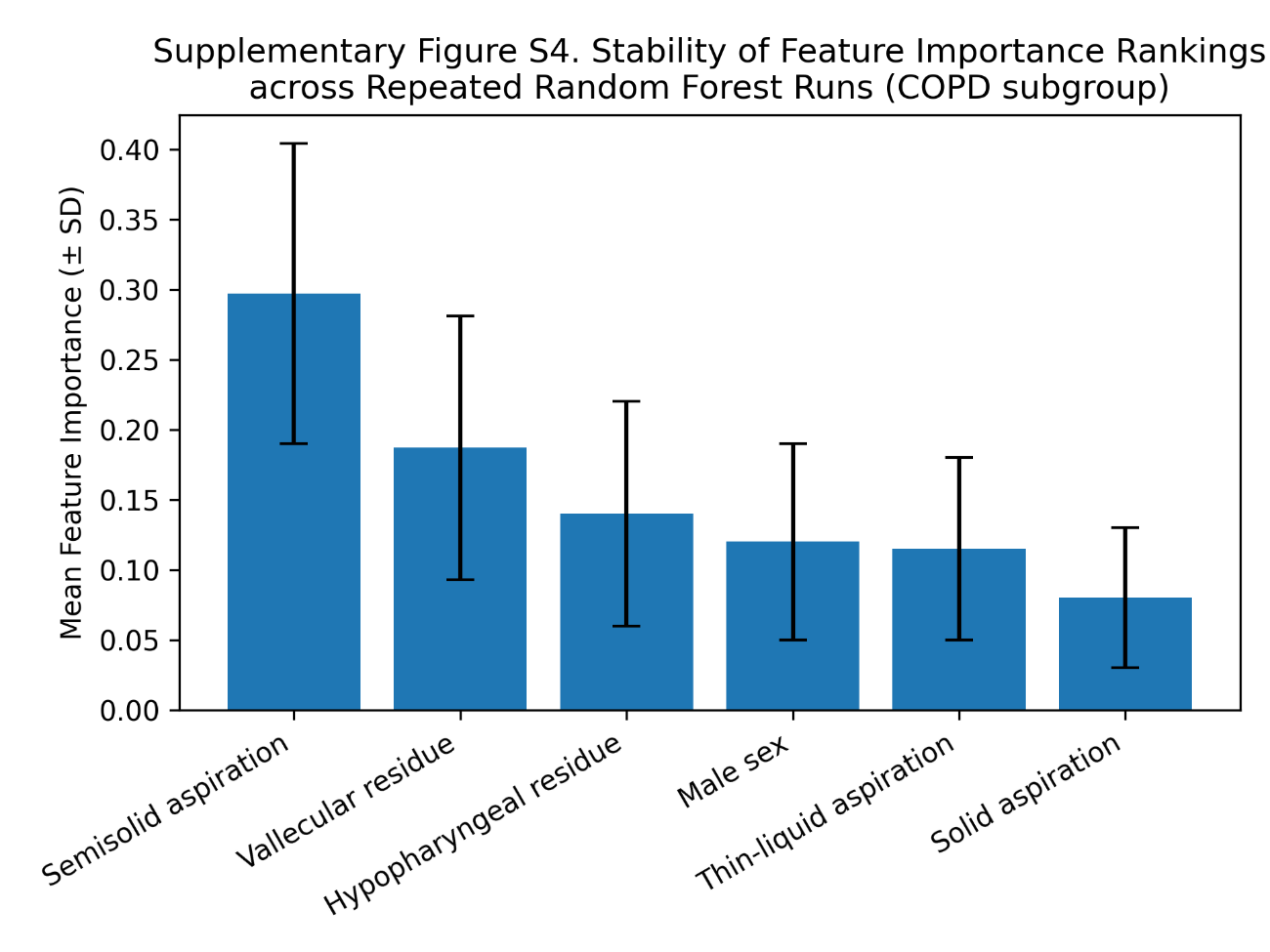
**

**Figure S4. Stability of feature importance rankings across repeated Random Forest runs in the COPD subgroup.** Mean feature importance values (± standard deviation) are shown across 200 repeated Random Forest runs using different random seeds. Semisolid aspiration demonstrated the highest and most stable importance, followed by vallecular residue. Other variables, including hypopharyngeal residue, male sex, and thin-liquid aspiration, showed intermediate stability, whereas solid aspiration ranked consistently lower. These results support the robustness of key predictors across internal resampling and indicate that their prioritization was not driven by a single model fit.

| **Predictor** | **Median OR (bootstrap)** | **95% CI (percentile)** | **% Positive Coefficient** | **Interpretation** |
| --- | --- | --- | --- | --- |
| Semisolid aspiration | 2.50 | 1.13 – 5.20 | 98.8% | Strong and highly stable association with aspiration |
| Vallecular residue | 1.49 | 0.70 – 3.26 | 85.4% | Moderate association with some variability |

**Supplementary Table S1A. Stability of logistic regression predictors in the COPD subgroup based on bootstrap resampling.** This table summarizes the robustness of predictors identified in the multivariable logistic regression model for aspiration (PAS ≥6) within the COPD subgroup. Odds ratios (ORs) and 95% confidence intervals were estimated using percentile bootstrap methods based on 2000 resampling iterations. Direction stability represents the proportion of bootstrap samples in which the regression coefficient remained positive. High direction stability indicates consistent association across resampled datasets, supporting the reliability of the identified predictors despite limited sample size.

| **Predictor** | **Mean Importance** | **SD Importance** | **Top-3 Frequency (%)** | **Stability Level** |
| --- | --- | --- | --- | --- |
| Semisolid aspiration | 0.297 | 0.110 | 91.5% | High |
| Vallecular residue | 0.187 | 0.088 | 61.0% | Moderate |

**Supplementary Table S1B. Stability of Random Forest feature importance rankings in the COPD subgroup across repeated model runs.**

This table reports the stability of variable importance derived from Random Forest models trained to predict aspiration (PAS ≥6) in the COPD subgroup. Feature importance values are expressed as mean ± standard deviation across repeated model runs using different random seeds. Top-3 frequency indicates the proportion of runs in which each variable ranked among the three most important predictors. This analysis evaluates the robustness of machine learning–based variable prioritization under internal resampling conditions.

| **Predictor** | **Regression Stability** | **RF Stability** | **Overall Evidence** |
| --- | --- | --- | --- |
| Semisolid aspiration | Very high | Very high | Strong |
| Vallecular residue | Moderate | Moderate | Consistent |

**Supplementary Table S1C. Integrated robustness of key predictors across regression and machine learning analyses in the COPD subgroup.**

This table provides a qualitative synthesis of predictor stability by integrating results from bootstrap-based logistic regression and Random Forest feature importance analyses. Regression stability reflects consistency of direction and magnitude of association across bootstrap iterations, while machine learning stability reflects reproducibility of variable importance rankings. The combined interpretation highlights predictors showing convergent evidence across analytical approaches, supporting their potential relevance despite the exploratory nature of subgroup analyses.

| **Variable** | **Bootstrap OR (95% CI)** | **Direction Stability (%)** | **RF Importance (mean ± SD)** | **Top-3 Frequency (%)** |
| --- | --- | --- | --- | --- |
| Semisolid aspiration | 2.50 (1.13–5.20) | 98.8 | 0.297 ± 0.110 | 91.5 |
| Vallecular residue | 1.49 (0.70–3.26) | 85.4 | 0.187 ± 0.088 | 61.0 |

**Supplementary Table S1D. Integrated stability of predictors in the COPD subgroup across bootstrap and machine learning analyses.**

Bootstrap OR values are derived from 2000 resampling iterations. Direction stability indicates the proportion of bootstrap samples in which the regression coefficient remained positive. Random Forest importance values are averaged across repeated runs with different random seeds. Top-3 frequency represents the proportion of runs in which the variable ranked among the three most important predictors.

| **Metric** | **Value** | **Interpretation** |
| --- | --- | --- |
| Brier score | 0.17 | Acceptable overall calibration and probabilistic accuracy |
| Calibration intercept | -0.05 | Minimal systematic overestimation of predicted risk |
| Calibration slope | 0.92 | Slight overfitting with modest shrinkage of predictions |
| AUC | 0.81 | Good discrimination performance |

**Supplementary Table S2. Calibration performance of the Random Forest model predicting aspiration (PAS ≥6).**The Brier score reflects the mean squared difference between predicted probabilities and observed outcomes, with lower values indicating better calibration. The calibration intercept evaluates systematic prediction bias, with values close to 0 indicating no global over- or underestimation. The calibration slope assesses the spread of predicted risks, with values close to 1 indicating appropriate calibration. AUC is reported for reference to discrimination performance.


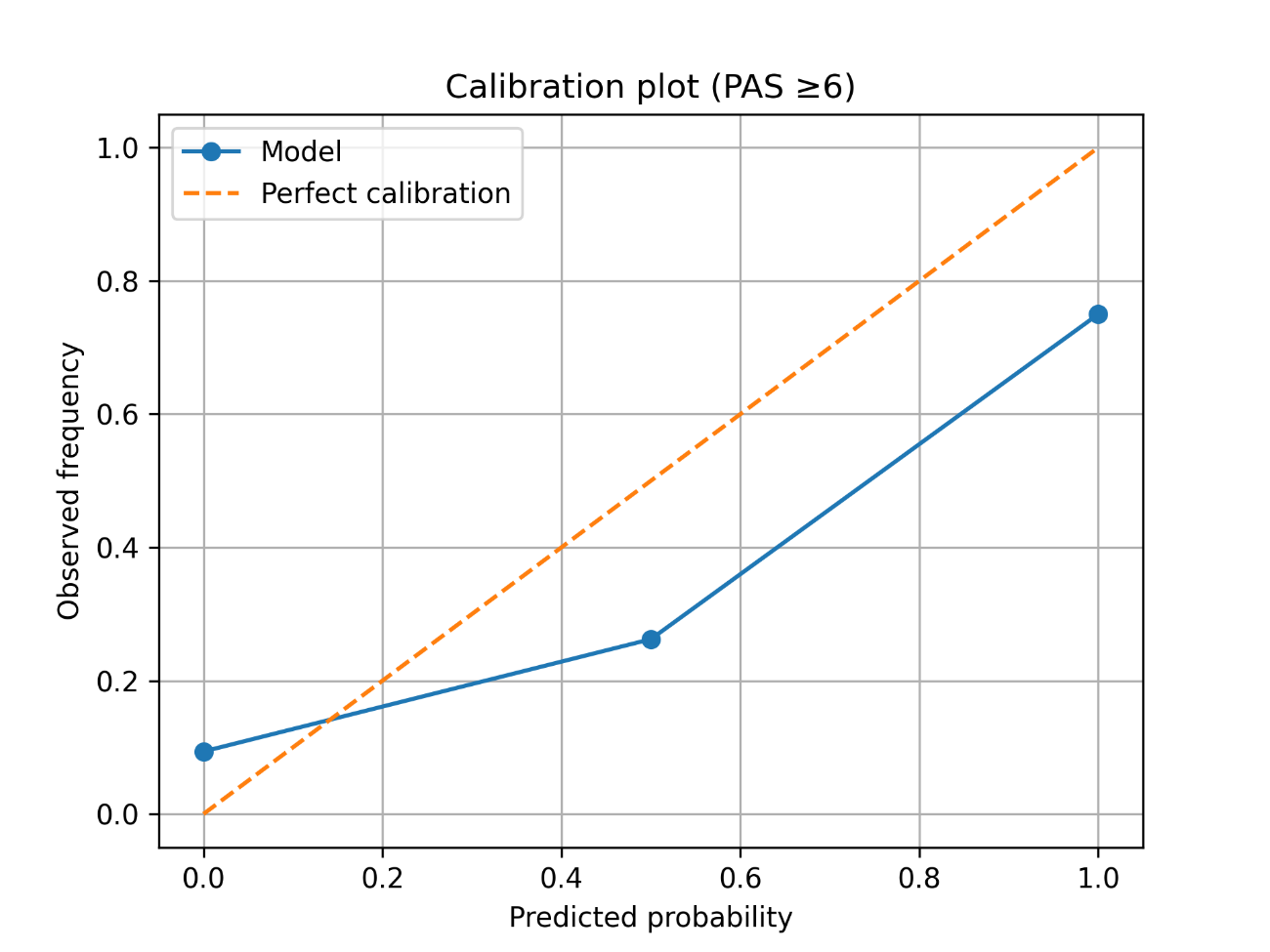


**Figure S4. Calibration plot of the Random Forest model predicting aspiration (PAS ≥6).** Calibration curve showing agreement between predicted and observed aspiration probabilities. The x-axis represents predicted probabilities of aspiration (PAS ≥6), and the y-axis represents observed event frequencies estimated using grouped or smoothed calibration. The diagonal dashed line indicates perfect calibration. The model demonstrates acceptable agreement across most probability ranges, with minor deviations at higher predicted risk levels consistent with slight overfitting.
